# Pretreatment Prediction of Individual Rheumatoid Arthritis Patients’ Response to Anti-Cytokine Therapy Using Serum Cytokine/Chemokine/Soluble Receptor Biomarkers

**DOI:** 10.1371/journal.pone.0132055

**Published:** 2015-07-15

**Authors:** Kazuko Uno, Kazuyuki Yoshizaki, Mitsuhiro Iwahashi, Jiro Yamana, Seizo Yamana, Miki Tanigawa, Katsumi Yagi

**Affiliations:** 1 Department of Organic Fine Chemicals, Division of Biological and Molecular Sciences, The Institute of Scientific and Industrial Research, Osaka University, Suita, Osaka, Japan; 2 Louis Pasteur Center for Medical Research, Kyoto, Kyoto, Japan; 3 Higashi Hiroshima Memorial Hospital, Higashi Hiroshima, Hiroshima, Japan; 4 Tokushukai Medical Corporation, Tokyo, Japan; Institute of Immunology, Rikshospitalet, NORWAY

## Abstract

The inability to match rheumatoid arthritis (RA) patients with the anti-cytokine agent most efficacious for them is a major hindrance to patients’ speedy recovery and to the clinical use of anti-cytokine therapy. Identifying predictive biomarkers that can assist in matching RA patients with more suitable anti-cytokine treatment was our aim in this report. The sample consisted of 138 RA patients (naïve and non-naïve) who were administered tocilizumab or etanercept for a minimum of 16 weeks as a prescribed RA treatment. Pretreatment serum samples were obtained from patients and clinical measures of their disease activity were evaluated at baseline and 16 weeks after treatment commenced. Using patients’ pretreatment serum, we measured 31 cytokines/chemokines/soluble receptors and used multiple linear regression analysis to identify biomarkers that correlated with patients’ symptom levels (DAS28-CRP score) at week 16 and multiple logistic analyses for biomarkers that correlated with patients’ final outcome. The results revealed that sgp130, logIL-6, logIL-8, logEotaxin, logIP-10, logVEGF, logsTNFR-I and logsTNFR-II pretreatment serum levels were predictive of the week 16 DAS28-CRP score in naïve tocilizumab patients while sgp130, logGM-CSF and logIP-10 were predictive in non-naïve patients. Additionally, we found logIL-9, logVEGF and logTNF-α to be less reliable at predicting the week 16 DAS28-CRP score in naïve etanercept patients. Multiple linear regression and multiple logistic regression analyses identified biomarkers that were predictive of remission/non-remission in tocilizumab and etanercept therapy. Although less reliable than those for tocilizumab, we identified a few possible biomarkers for etanercept therapy. The biomarkers for these two therapies differ suggesting that their efficacy will vary for individual patients. We discovered biomarkers in RA pretreatment serum that predicted their week 16 DAS28-CRP score and clinical outcome to tocilizumab therapy. Most of these biomarkers, especially sgp130, are involved in RA pathogenesis and IL-6 signal transduction, which further suggests that they are highly reliable.

**Trial Registration:**

UMIN-CTR Clinical Trial UMIN000016298

## Introduction

Modern research in rheumatic disease has led to the development of biopharmaceutical products that are used in anti-cytokine therapies that target rheumatoid arthritis (RA). Among such RA anti-cytokine therapies infliximab, etanercept, adalimumab, golimumab, certolizumab and tocilizumab are the most widely used clinically. Currently, anti-TNF-α and anti-IL-6 agents are the standard treatment for RA[1–8]. Based on prior studies, the average patient remission rate varies according to the treatment involved and fall between 17 to 59% in patients naïve to anti-IL-6 therapy with/without methotrexate (MTX)[9–12] and 21 to 46% in patients naïve to anti-TNF-α agents with/without MTX [1–3,7,13–16]

In clinical practice it has been noted that each anti-rheumatic therapy delivers a different outcome for individual RA patients and this makes it difficult to prescribe the most efficacious treatment for them. Being able to predict a patient’s response/outcome before they are treated would allow doctors to prescribe the cytokine therapy that is the most efficacious for each RA patient. This would result in time and cost benefits, improvements in the quality of life for RA patients and reduce the risk of patients experiencing disability from long-term joint damage. To this end, it is critical to identify molecular biomarkers that can predict patient response to anti-TNF-α or anti-IL-6 based therapies before patients are treated so that non-effective therapies are eliminated and more effective ones can be prescribed for patients at an earlier stage.

The European League Against Rheumatism (EULAR) has proposed remission as the ultimate target in the treatment of RA, with low disease activity (low DAS score) being an alternate goal in patients who cannot achieve remission or who fail to sustain remission. Additionally in 2010, EULAR recommended a treat-to-target approach for RA therapy and the American College of Rheumatology [17,18] later accepted this recommendation. We believe that identifying reliable predictive biomarkers will make it easier to follow EULAR’s treat-to-target recommendation by allowing clinicians to know in advance if a treatment strategy will achieve the treatment goal (target) that has been pre-determined for each RA patient.

Recently a number of trails to find candidate biomarkers are being conducted through genomic, proteomic and cytokine/chemokine analysis [19–22], and several reports have identified predictive markers, however most of them can only be applied during treatment to decide if patients should continue treatment or not. While a few reports have used proteomic analysis [23,24], genome micro-array[25] and serum markers including several cytokines[26], to identify biomarkers to predict treatment outcome to anti-TNF-α therapy, only two recent reports have identified biomarkers to predict RA outcome to anti-IL-6 therapy using sIL-6R levels [27] or genome micro-array [28]. Most studies are unable to predict patient outcome or response prior to them undergoing treatment and performing genome based studies for all patients is prohibitive due to ethical issues, the high cost involved and the need for patient’s gene sample. We believe that making a prediction before therapy using blood serum may be possible because patients’ serum cytokine/chemokine levels tend to reflect patients’ disease and genetic status. In this study we used a Luminix beads based array method to measure and analyze cytokines/chemokines simultaneously in RA patient’s pretreatment serum to identify biomarkers that could predict each patient’s response and outcome before therapy.

It was recently shown that soluble IL-6 receptor (sIL-6R) enhanced IL-6 activity in the rheumatoid synovium, but that the development of arthritis in various model systems could be blocked by soluble gp130Fc (sgp130Fc) [29,30]. Other studies have highlighted the importance of sIL-6R levels in anti-IL-6 therapy [27] therefore we measured soluble receptors related to the IL-6 signal pathway in addition to cytokines and chemokines.

In this retrospective observational cohort study, we analyzed pretreatment serum samples and data for a cohort of RA patients and used multiple linear regression analysis to reveal biomarkers that predicted RA patients’ week 16 DAS28-CRP score to tocilizumab or etanercept therapy. Multiple logistic regression analysis revealed whether or not patients would achieve remission at week 16 (this is the time frame when therapeutic efficacy is usually judged and doctors decide if patients should continue or discontinue treatment).

## Patients and Methods

### Patients

This study consisted of 138 patients who were previously diagnosed with RA (1990 ACR criteria) and were consecutively administered at Higashi Hiroshima Memorial Hospital (Hiroshima, Japan) between March 2008 and June 2013. Patients underwent treatment with tocilizumab or eternacept. Tocilizumab treated patients totaled 88; 48 patients were considered as biologic naïve and 40 patients were non-naïve. The etanercept treated group consisted of 43 biologic naïve and 7 non-naïve patients. Biologic naïve patients are those who failed prior treatments using disease modifying antirheumatic drugs (DMARDs) but had not undergone cytokine therapy, while non-naïve patients are those who failed one to three prior treatments with anti-TNF-α and methotrexate therapy.

**Fig 1:**
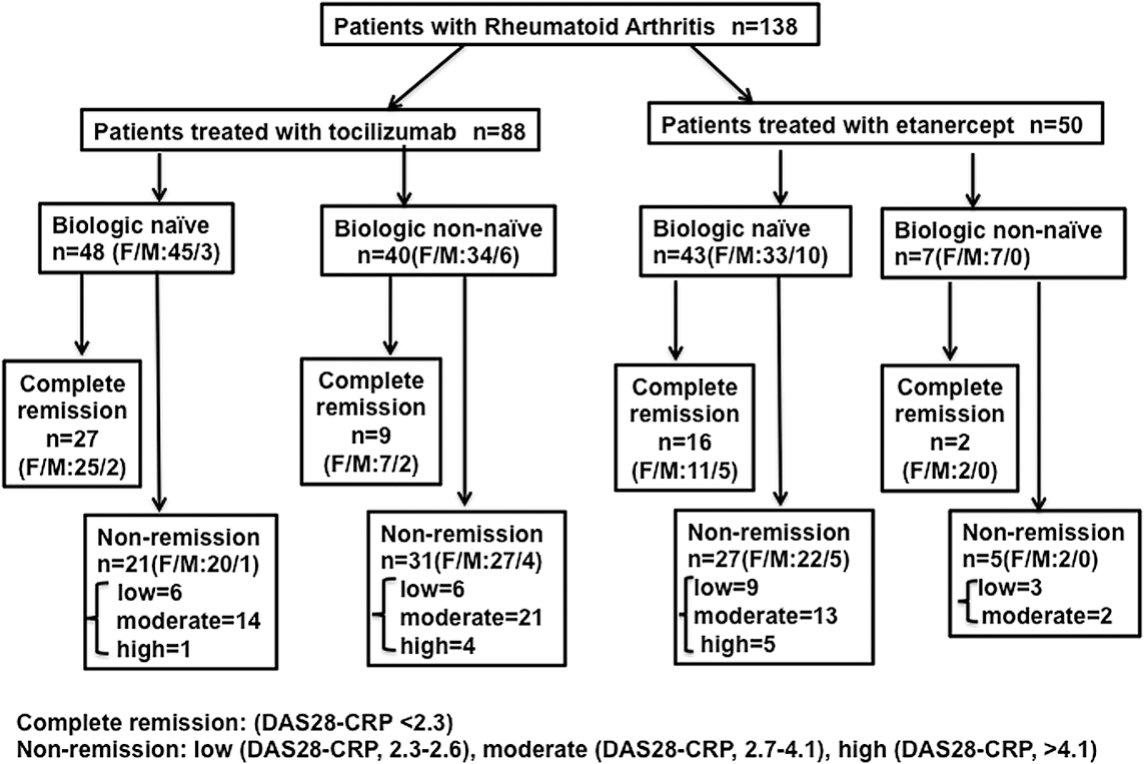
Sample profile and patient outcome to tocilizumab therapy. Patient’s final outcome was based on their DAS28-CRP score 16 weeks after the first tocilizumab or etanerecept treatment. Non-naïve patients are those who had prior anti-cytokine treatment one to three times.

Table 1 summarizes the baseline clinical characteristics of patients, while Fig 1 shows the trial profile of patients who underwent tocilizumab or etanercept therapy. For the purpose of this observational study, we selected patients who underwent at least 16 weeks of treatment, had serum and clinical data that were available for analysis when this study commenced in 2008, and those who did not develop adverse reactions to treatment.

**Table 1:** Baseline profile of tocilizumab and etanercept patients.

		Biologic naïve patients treated with tocilizumab	Non-naïve patients treated with tocilizumab	Biologic-naive patients treated with etanercept
Clinical parameters	Number of patients (Female/Male)	n = 48 (F/M:45/3)	n = 40 (F/M:34/6)	n = 43 (F/M:33/10)
Age	year	59.4±1.8*	56.8±1.8	59.2±1.9
Duration of disease	year	10.6±1.2	10.9±1.2	7.9±8.4
WBC	x 10^3^/ μl	8254±424	8192±371	8173±456
Fe	mg/dl	41.5±4.5	57.5±7.3	54.2±5.4
Ferritin	ng/dl	94.8±12.8	64.3±9.1	140.1±181.8
RBC	x 10^6^/ μl	384.2±8.4	409.8±8.7	402.9±8.1
Hb	g/dl	11.0±0.2	11.8±0.3	11.9±0.2
Ht	%	35.4±0.6	37.6±0.8	37.3±0.7
Plt	x 10^3^/ μl	32.3±1.3	29.0±1.4	29.7±1.5
CRP	mg/dl	3.5±0.7	2.4±0.5	2.8±0.4
DAS28-CRP		4.6±0.2	4.4±0.1	4.7±0.2
RF	U/ml	155.0±31	89.2±14.5	188.3±53.2
VAS	mm	53.3±3.7	57.3±3.6	57.1±4.3
MMP-3	ng/ml	322.9±38.2	275.5±42.9	248.9±30.9
Swollen joint count		7.0±0.9	5.1±0.5	6.4±0.7
Tender joint count		6.3±0.9	5.3±0.4	6.6±0.8
Stage		2.9±0.2	3.7±0.1	2.8±0.2
Class		2.0±0.1	2.3±0.1	2.2±0.1

*Values are the mean ± SEM

WBC: white blood cells, RBC: red blood cells, Hb: hemoglobin, Ht: hematocrit, Plt: platelet count, CRP: C-reactive protein, DAS28-CRP: disease activity acore 28 C-reactive protein, RF: rheumatoid factor, VAS: visual analog scale, MMP-3: matrix metalloproteinase protein 3

To create a control baseline cytokine level, we analyzed blood serum for healthy subjects (n = 51, Female:n = 34, 45.1± 2.3/Male:n = 17, 47.9±3.4) who had given blood samples during standard routine health checks at Louis Pasteur Center for Center for Medical Research. This baseline was used to determine the normal healthy distribution pattern of each cytokine/chemokine/ soluble receptor. Healthy subjects had no history of chronic inflammatory diseases including RA, viral hepatitis or cancer. Healthy subjects and patients gave their written informed consent before providing the hospital with blood samples. Ethical approval was obtained from the Higashi Hiroshima Memorial Hospital Ethical Committee (permission HMH-09-05). This study is registered in the University Hospital Medical Information Network as a non-interventional retrospective observation study with the identifier University Hospital Medical Information Network (UMIN) 000016298.

### Study procedures

We assessed tocilizumab and eternacept patients’ week 16 data to judge clinical efficacy based on patients’ DAS28-CRP score and whether patients experienced remission or non-remission. Naïve and non-naïve RA patients were treated with 8mg/kg of tocilizumab once every 4 weeks with or without MTX or were administered 25 mg of etanercept by subcutaneous injection once or twice a week with MTX. All patients had provided blood serum prior to receiving tocilizumab/etanerecept treatment. Using this pretreatment serum, we measured patients’ cytokine, chemokine and soluble receptor levels and selected as parameters those that correlated with patients’ disease activity score in 28 joints (DAS28-CRP) at week 16 of therapy using multiple linear regression analysis. DAS28-ESR is widely used to monitor disease activity in RA, however, it has been reported that DAS28-CRP (a grading system verified by Inoue et al.) and DAS28-ESR are interchangeable and produce similar results [31]. Although DAS28-ESR data is not available in all patients, comparison of DAS28-CRP and DAS28-ESR were well co-related as shown in S1 Fig.

Since it is standard for clinicians to judge whether patients experience remission or non-remission before altering treatment, we determined patient’s remission status. While a DAS28-CRP score of < 2.6 is the standard considered as remission, we used a stricter limit of DAS28-CRP < 2.3 in this study. Non-remission RA patients were allocated into three groups based on the severity of their symptoms as reflected by their DAS28-CRP score: low (2.3–2.6), moderate (2.7–4.1), and high (>4.1). The same doctor at Higashi Hiroshima Memorial Hospital determined the clinical outcome of all patients at week 16 and this potentially eliminates any bias.

### Cytokines/chemokines/soluble receptors assay

A total of 138 serum samples from 138 patients were analyzed before therapy and at 16 weeks from the start of treatment. At the time blood sera from RA patients and healthy subjects were collected they were centrifuged at 1600 g for 10 min. These serum samples were kept frozen at −80°C until they were made available to be analyzed for this study. We simultaneously quantified 31 cytokines/chemokines /soluble receptors in RA and healthy subjects’ serum to determine their distribution pattern using Bio-Plex 200, a multiplex cytokine array system (Bio-Rad Laboratories, CA, USA) according to the manufacturer's instructions. The Bio-Plex Human Cytokine 27-Plex Panel includes 27 cytokines and chemokines (IL-1β, IL-1Ra, IL-2, IL-4, IL-5, IL-6, IL-7, IL-8, IL-9, IL-10, IL-12 (p70), IL-13, IL-15, IL-17, basic FGF, eotaxin, G-CSF, GM-CSF, IFN-γ, IP-10, MCP-1, MIP-1α, MIP-1β, PDGF-bb, RANTES, TNF-α, VEGF). SIL-6R, sgp130, sTNFR-I and sTNFR-II (Milliplex^R^ MAP, Human Soluble Cytokine Receptor Panel: Millipore Co., MA, USA) were also measured for a total of 31. Data acquisition and analysis were performed using Bio-Plex Manager software version 5.0.

### Statistical analysis

The distribution of cytokine/chemokine/soluble receptor values in healthy controls was analyzed to determine whether the raw values or log-transformed values were more normally distributed. All parameters except sgp130 had log-transformed values that were more normally distributed (data not shown), and so they were used in our analysis. Cytokine/chemokine/soluble receptor values are expressed as pg/ml except sgp130 which is expressed as μg/ml. For each group of patients, multiple linear regression analysis and multiple logistic regression analysis were performed. As the number of non-naïve patients treated with etanercept was low, we excluded this group from the analysis. Simple and multiple linear regression analysis were used to determine if any relationship existed between pretreatment cytokine/chemokine/soluble receptor levels and patients’ week 16 DAS28-CRP score. These values and clinical variables underwent a stepwise multiple linear regression analysis. The resulting parameters with p<0.05 were considered significant.

Since clinical doctors first observe if patients experience remission (DAS28-CRP below 2.3) or non-remission before switching patients’ treatment protocol, we performed multiple logistic regression analysis to determine patient’s remission status. All statistical analyses were carried out with JMP 9.0 software.

## Results

### Clinical assessment

**Fig 2:**
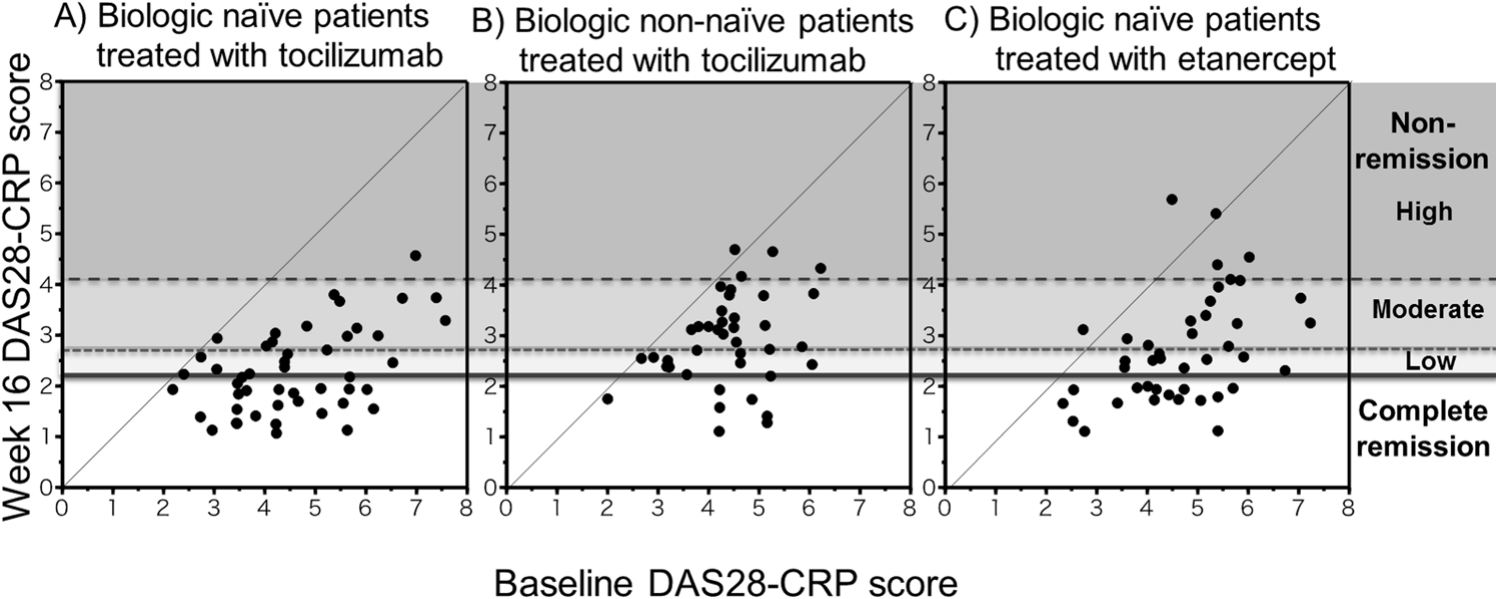
Scatter plot and regression lines for baseline and week 16 DAS28-CRP score in RA patients administered with tocilizumab (A: naïve and B: non-naïve) or etanerecept (C: naïve) therapy. Black circle to the left of the 45° line in Fig 2B represents the only non-naïve tocilizumab patient with no improvement in their DAS28-CRP score at week 16. Three black circles to the left of the 45° line in Fig 2C represents etanerecept patients with no improvement in their DAS28-CRP score at week 16.

**Fig 3:**
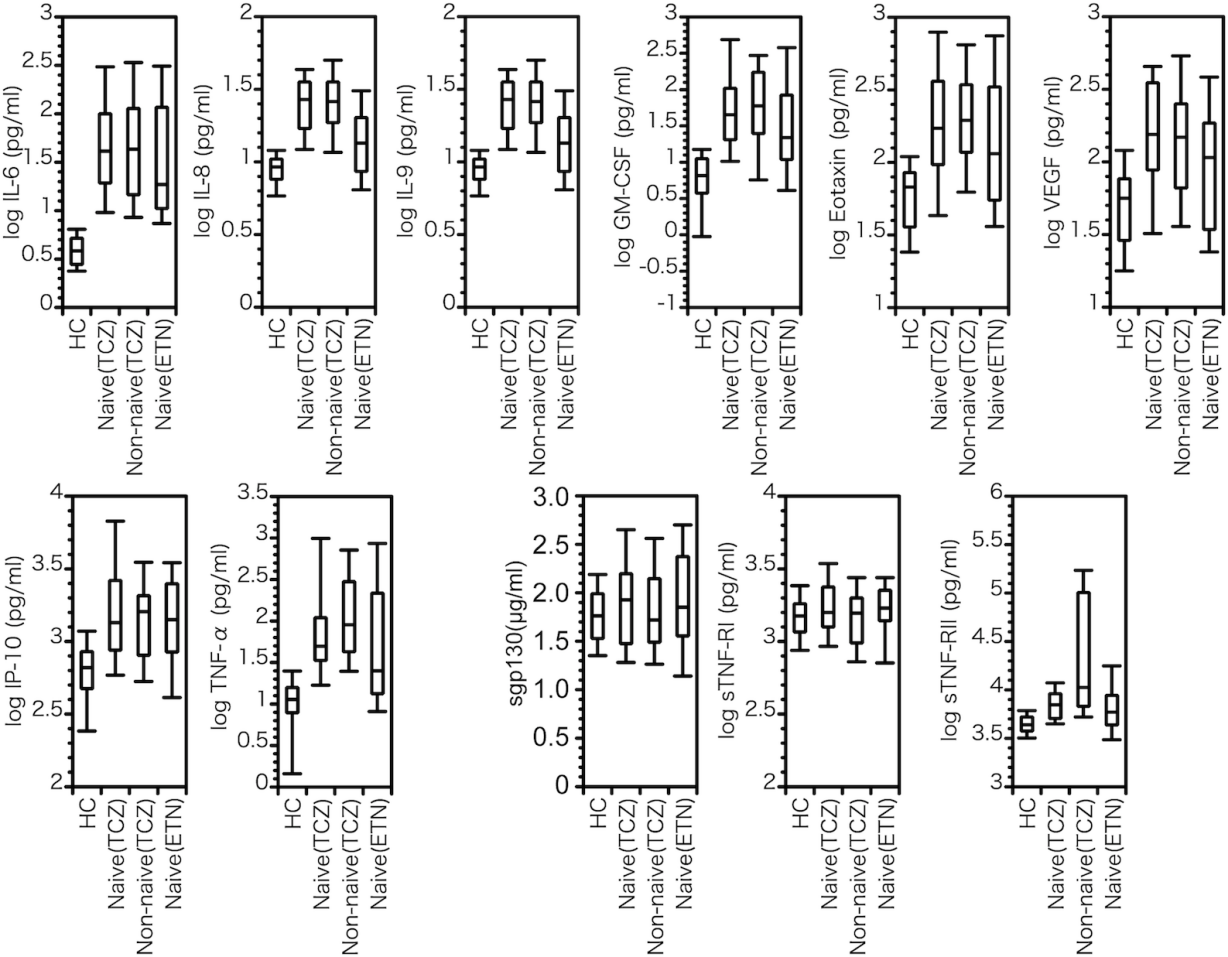
Box plot of typical cytokine/chemokine/soluble receptor levels in healthy controls (HC), tocilizumab treated naïve patients (Naïve (TCZ)), tocilizumab treated non-naïve patients (Non-naïve (TCZ)) and etanercept treated naïve patients (Naïve (ETN)). Sgp130 is expressed in raw values (μg/ml); the other variables are expressed in log-transformed values. Box plots consist of the minimum, first quartile, median, third quartile, and maximum.

Table 1 shows patients’ clinical baseline profile and Fig 2 shows their actual baseline and week 16 DAS28-CRP score. A comparison of cytokine/chemokine/soluble receptor baseline values in healthy subjects and RA patients of each group is shown in Fig 3. It is evident that most cytokine/chemokine levels in RA were significantly higher than in healthy subjects except for sgp130, sIL-6R sTNFR-I and sTNFR-II. In naïve and non-naïve patients, cytokine/chemokine/soluble receptor levels were relatively similar except for sTNFR-II which was higher in non-naïve patients treated with tocilizumab.

In naïve tocilizumab patients, 56% (n = 27) experienced clinical efficacies that were judged as complete remission, while the remaining 21 experienced non-remission (Fig 1). This remission rate was similar for patients whose treatment was combined with MTX (total n = 14, remission: 57%) and those treated with tocilizumab only (total n = 29, remission: 53%). At week 16, 9 out of 40 non-naïve tocilizumab patients experienced clinical efficacies that were judged as complete remission and the remaining 31 patients experienced non-remission. All tocilizumab patients except one non-naïve, showed improvements in their DAS28-CRP score at week 16 (Fig 2A and 2B). On the other hand, 37% (n = 16) of etarercept naïve patients experienced clinical efficacies that were judged as complete remission, while the remaining 27 experienced non-remission (Fig 2C). All except three naïve etanerecept patients showed improvements in their DAS28-CRP score at week 16.

### Predicting week 16 DAS28-CRP score using pretreatment serum cytokine/chemokine/soluble receptor levels

In this study, most patients treated with tocilizumab or etanercept showed improvement in their DAS28-CRP score at week 16 of therapy (Fig 2A, 2B and 2C). With this in mind, we attempted to predict the week 16 DAS28-CRP score for RA patients using their pretreatment cytokine/chemokine/soluble receptor data.

To find biomarkers that may have contributed to the week 16 DAS28-CRP score, we performed single linear regression analysis of the week 16 DAS28-CRP score as an objective variable using the raw or log transformed cytokine/chemokine/soluble receptor values and other clinical parameters as independent variables. We found that sgp130 significantly coincided with the week 16 DAS28-CRP score in naïve tocilizumab patients, while in non-naïve patients, sgp130, logIL-1β, logIL-2, logIL-5, logIL-15, logGM-CSF, logIFN-γ and logTNF-α significantly coincided with the week 16 DAS28-CRP score. On the other hand, logIL-9 significantly coincided with the week 16 DAS28-CRP score in naïve etanercept patients (data not shown).

Multiple linear regression analysis of cytokine/chemokine/soluble receptor levels was performed to determine the best equation of DAS28-CRP improvement. We found that sgp130, logIL-6, logIL-8, logEotaxin, logIP-10, logVEGF, logsTNFR-I and logsTNFR-II values were significantly expressed in naïve tocilizumab patients (R^2^ = 0.646, p<0.0001) (Table 2). As well, when logVEGF was excluded as a variable, sgp130, logIL-6, logIL-8, logEotaxin, logIP-10, logsTNFR-I and logsTNFR-II values were also significantly expressed in these naïve tocilizumab patients (R^2^ = 0.605, p<0.0001). Furthermore, logIL-1β (R^2^ = 0.595, p<0.0001) or logMCP-1 (R^2^ = 0.578, p<0.0001) in addition to sgp130, logIL-8, logEotaxin, logIP-10, logsTNFR-I and logsTNFR-II were substitutes for log IL-6 as a predictive biomarker. In non-naïve tocilizumab patients, we observed that sgp130, logGM-CSF and logIP-10 values were possible predictive biomarkers (R^2^ = 0.486, p<0.0001) (Table 2). A comparison of multiple linear regression analysis results for naïve patients in MTX with tocilizumab treated group and the tocilizumamb only treated group showed similar tendencies as the entire naïve tocilizumab population (S1 Table).

**Table 2:** Multiple linear regression analysis of week 16 DAS28-CRP score using cytokine/chemokaine/soluble receptor levels.

	Biologic naïve patients treated with tocilizumab		Non-naïve patients treated with tocilizumab		Biologic-naive patients treated with etanercept	
Number of patients (Female/Male)	n = 48 (F/M:45/3)		n = 40 (F/M:34/6)		n = 43 (F/M:33/10)	
R^2^	0.646		0.486		0.247	
p value	p<0.0001		p<0.0001		p = 0.0107	
Cytokine/Chemokine/soluble receptor	Estimate	p value	Estimate	p value	Estimate	p value
intercept	6.91	0.001	2.84	0.011	0.75	0.318
sgp130	-5.34	0.002	-6.04	0.003	-	-
logIP-10	-1.00	0.002	0.71	0.041	-	-
logIL-6	0.74	0.002	-	-	-	-
logIL-8	3.94	< .0001	-	-	-	-
logEotaxin	-1.04	< .0001	-	-	-	-
logsTNFRI	-2.58	< .0001	-	-	-	-
logsTNFRII	1.41	0.030	-	-	-	-
logVEGF	-0.85	0.039	-	-	0.76	0.070
logGM-CSF	-	-	-0.62	0.0003	-	-
logIL-9	-	-	-	-	0.67	0.044
logTNF-α	-	-	-	-	-0.53	0.048

-: excluded from analysis

sgp130: soluble gp130, IP-10: interferon gamma-induced protein 10, IL: interleukin, sTNFRI: soluble tumor necrosis factor receptor one, sTNFRII: soluble tumor necrosis factor receptor two, VEGF: vascular endothelial growth factor, GM-CSF: granulocyte macrophage colony-stimulating factor, TNF: tumor necrosis factor

Although our study sample was small, multiple linear regression analysis of cytokine/chemokine/soluble receptor levels using the week 16 DAS28-ESR clearly showed that sgp130, logIL-6, logIL-8, logEotaxin, logIP-10 values were possible predictive biomarkers in biologic naïve patients (p = 0.0003) and sgp130, logGM-CSF and logIP-10 values were possible predictive biomarkers in non-naïve patients (p<0.0001). (S2 Table)

Although the R^2^ value was not sufficiently high (albeit significant), we observed that logIL-9, logTNF-α and logVEGF values were possible predictive biomarkers in naïve etanercept patients (R^2^ = 0.247, p = 0.0107) (Table 2). The predictive biomarkers we identified for tocilizumab and etanercept therapy are quite different therefore using these biomarkers to determine which treatment will be more effective for each patient can deliver great benefits for the patients involved.

### Predicting patients’ week16 outcome using pretreatment serum cytokine/chemokine/soluble receptor levels

Multiple linear regression analysis showed that it was possible to predict patients’ week 16 DAS28-CRP score. Since clinical doctors cannot make this prediction beforehand they often wait for patients to experience remission or non-remission before altering patients’ treatment. Cytokine/chemokine/soluble receptor data for RA patients were analyzed using single logistic analysis and a comparison was done for the remission and non-remission groups. Single logistic regression analysis showed that sgp130 was significantly different between biologic naïve and non-naïve patients who experienced remission versus those who were in non-remission. Additionally, logsIL-6R was significantly different between remission and non-remission in non-naïve patients. On the other hand, logIL-9 was significantly different between naïve etanercept patients who experienced remission and those who did not (data not shown).

**Fig 4:**
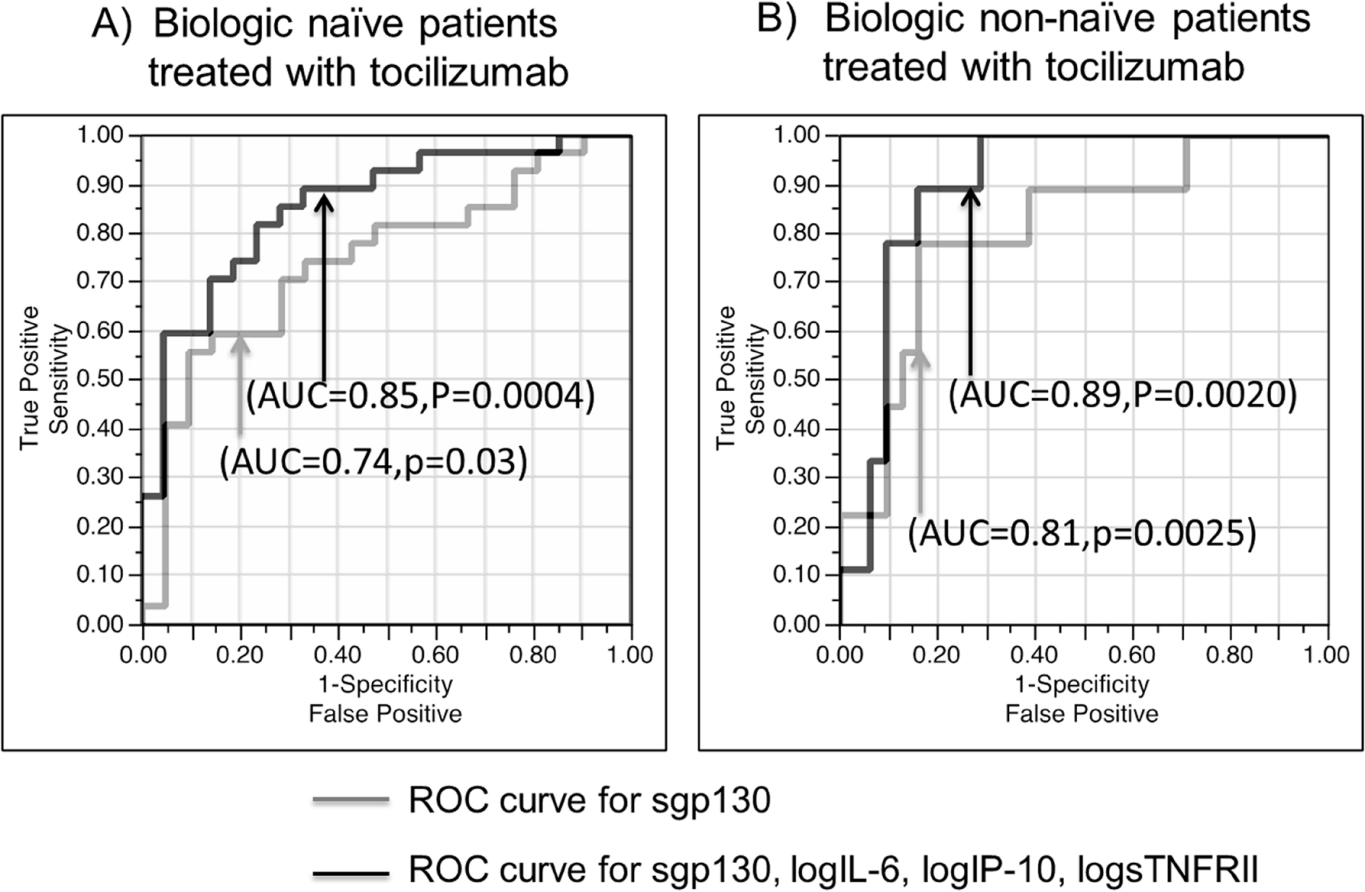
ROC curves for predicting remission in biologic naïve patients (A) and non-naïve patients (B) to tocilizumab therapy. ROC curve for sgp130 (gray line); ROC curve for sgp130, logsTNFR-II, logIP-10 and logIL-6 (black line).

Multiple logistic regression analysis was used to determine multivariable models as predictive biomarkers of remission and non-remission based on baseline cytokine/chemokine/soluble receptor levels in biologic naïve tocilizumab patients. The best combination of predictive markers is shown in Table 3. The data strongly suggests that sgp130, logIL-6, logIP-10, and logsTNFR-II values are potential predictive biomarkers both naïve (p = 0.0004, AUC = 0.850, Fig 4A) and non-naïve patients (p = 0.002, AUC = 0.892 Fig 4B). Furthermore, logIL-7 (p = 0.0003, AUC = 0.848), logIL-1β (p = 0.0005, AUC = 0.853) or logMCP-1 (p = 0.0004, AUC = 0.848) were substitute predictive biomarkers for logIL-6 in naïve patients and logIL-1β (p = 0.002, AUC = 0.899) in non-naïve patients (data not shown). Fig 4A and 4B, shows ROC curve of sgp130 in naïve (p = 0.0030, AUC = 0.741, Fig 4A) and non-naïve cases (p = 0.0025, AUC = 0.81,Fig 4B). These results show that sgp130 is a key predictive biomarker in naïve and non-naïve tocilizumab patients.

**Table 3:** Multiple logistic regression analysis of remission using cytokine /chemokaine/soluble receptor levels.

	Biologic naïve patients treated with tocilizumab		Non-naïve patients treated with tocilizumab		Biologic-naive patients treated with etanercept	
Number of patients (Female/Male)	n = 48 (F/M:45/3)		n = 40 (F/M:34/6)		n = 43 (F/M:33/10)	
p value	p = 0.0004		p = 0.0020		p = 0.0115	
Cytokine/Chemokine/soluble receptor	Estimate	p value	Estimate	p value	Estimate	p value
intercept	-5.09	0.466	-10.94	0190	-1.00	0.337
sgp130	-36.65	0.001	-29.05	0.020	-	-
logIP-10	-4.00	0.007	4.47	0.060	-	-
logIL-6	1.66	0.034	-2.76	0.047	-	-
logsTNFRII	5.63	0.016	2.07	0.084	-	-
logIL-9	-	-	-	-	1.71	0.012
logTNF-α	-	-	-	-	-1.03	0.079

-: excluded from analysis

sgp130: soluble gp130, IP-10: Interferon gamma-induced protein 10, IL: Interleukin, sTNFRII: soluble tumor necrosis factor receptor two, TNF: Tumor necrosis factor

**Fig 5:**
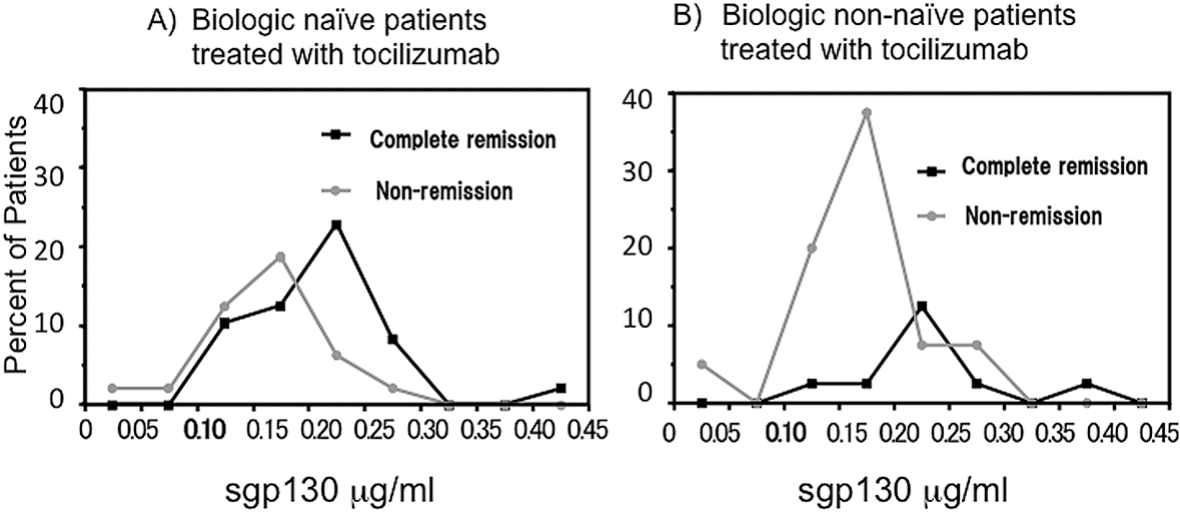
Differential distributions of pretreatment serum soluble gp130 levels in biologic naïve patients (remission:black line, non-remission:gray line). Serum soluble gp130 levels (μg/ml) were determined before therapy and remission was considered as DAS28-CRP score of 2.3 and below after 16 weeks of therapy.

Fig 5A and 5B show the distribution for tocilizumab remission and non-remission patients according to their pretreatment serum sgp130 levels. Among naïve patients 59.2% of those who experienced remission and 19.0% of non-remission patients showed sgp130 levels over 0.2 μg/ml. Among non-naïve patients 66.6% of remission and 19.3% of non-remission patients had sgp130 levels exceeding 0.2 μg/ml. These results suggest that sgp130 is an important predictor of RA patients’ clinical outcome to tocilizumab therapy.

On the other hand, AUC value was low, logIL-9 and logTNF-α ware significant possible predictive biomarkers (p = 0.0115, AUC = 0.745) for etenercept therapy (Table 3). It is important to note that the biomarkers that predicted remission or non-remission in tocilizumab are different from those for etanercept.

## Discussion

In this retrospective observational analysis, we used pretreatment serum soluble receptor and cytokine/chemokine levels to identify reliable biomarkers to predict the week 16 DAS28-CRP score and remission/non-remission in RA patients who were administered tocilizumab or eternacept. Our analysis based on DAS28-CRP as an objective variable revealed that pretreatment sgp130, logIL-6, logIL-8, logEotaxin, logIP-10, logVEGF, logsTNFR-I and logsTNFR-II levels were predictive of naïve tocilizumab patients’ week 16 DAS28-CRP and sgp130, logGM-CSF and logIP-10 were predictive of non-naïve patients’ DAS28-CRP score. Although reliability is a little low, logIL-9, logTNF-α and logVEGF levels were predictive of the week 16 DAS28-CRP score in etanercept patients. It is an important finding that biomarkers that can predict RA’s week 16 DAS28 score are completely different for tocilizumab and etanercept; we believe this suggests that the therapeutic mechanism of each anti-cytokine agent is different.

In analyzing data that represented patient’s week 16 clinical outcomes, we discovered that sgp130, logIL-6, logIP-10 and logsTNFR-II were significant markers to predict if naïve RA patients would experience remission or not post tocilizumab therapy. Among these factors, a high sgp130 level was the most predictive (Table 3, Fig 4A and 4B, Fig 5A and 5B). For patients treated with etanercept, it was apparent that logIL-9 and logTNF-α were predictive of remission or non-remission. Here also the remission/non-remission biomarkers were different from tocilizumab and etanercept.

We believe that the predictive biomarkers we identified through quantifying cytokines/chemokines/soluble receptors are more practical and useful than gene analysis. These biomarkers can be measured using as little as 100μl of patients’ pretreatment blood serum. It is more difficult to obtain ethical approval for DNA micro-array to find biomarkers, and the array is also expensive. Additionally, gene analysis requires that RNA is quickly extracted after blood sample is drawn; this means that only a few clinics are able to prepare test samples to identify predictive biomarkers for RA. On the other hand, identifying serum biomarkers require only a small amount of patients’ blood serum before therapy so it is possible to use residual serum after a standard blood test. Determining pretreatment serum biomarkers for individual patients in this way allows patients to have more targeted treatments that will deliver better outcomes for RA patients.

To test this idea, we created a prediction model for the scenario that etanercept patients were instead treated with tocilizumab. We found that half of those patients would have achieved a better outcome with tocilizumab, one third would have gotten the same results, and several patients would have had a worse (higher) DAS28-CRP score from tocilizumab therapy (data not shown). These results provide evidence that each patient responds differently to each anti-cytokine treatment and may respond more favorably to one over another.

The role of all biomarkers identified for tocilizumab or etanercept therapy are difficult to thoroughly explain through their mechanism. They are inflammatory cytokines/chemokines and signal related soluble receptors. Clinical and laboratorial improvements in IL-6 blocking therapy have led to a decrease in inflammation in RA. This results from the reduction in acute phase proteins in patients’ serum such as CRP and SAA, and an increase in albumin, which leads to an improvement in inflammatory anemia via hepcidin. This at least suggests that IL-6, IL-1 and TNF-α in some way contribute to the induction of CRP, SAA and hepcidin in RA [32–34] and that the production of VEGF is enhanced by the aforementioned cytokines [35].

In regards to the IL-6 receptor system, sIL-6R increases in the presence of inflammation. IL-6 and sIL-6R complex activates a wide variety of cells that express gp130 on their cell membrane in inflammatory conditions suggesting that sIL-6R and related soluble receptors contribute to IL-6 signaling and may be predictive markers for patient outcome to tocilizumab therapy.

Sgp130 in particular was especially highly predictive; high levels before therapy was a reliable marker of favorable outcome to tocilizumab therapy. We believe that sgp130 may support the inhibition of IL-6 activity in RA patients being treated with tocilizumab. Fig 5 supports this notion; most patients with serum sgp130 levels above 0.2 μg/ml experienced remission after 16 weeks of tocilizumab therapy, but patients with lower sgp130 levels did not. Sgp130, which is secreted when the gp130 gene is spliced, is a naturally occurring antagonist of the IL-6/sIL-6R complex. Stefan Rose et al. [36] previously reported that when sgp130 levels are high, more IL-6/IL-6R complex are neutralized by sgp130 and less free IL-6/IL-6R are left in the serum to be neutralized by tocilizumab [36]. This may explain our observation that patients with higher sgp130 levels were prone to experience higher clinical efficacy to tocilizumab therapy. We therefore believe that in order to find beneficial clinical parameters, it is necessary to analyze the signal pathways of cytokines and their soluble receptors related to the pathogenesis of RA.

Although logIL-9, logTNF-α and logVEGF were predictive of etanercept patients’ week 16 DAS28-CRP score, their reliability was lower than for tocilizumab. Finding more reliable markers is essential so that etanercept therapy can be used more efficiently and effectively. Further research can result in the discovery of new biomarkers that can more accurately predict patient outcome to etanercept therapy. Our ultimate aim is to develop a kit that can help clinicians to choose the anti-cytokine agent that is most suitable for individual patients before administering treatment.

We believe that it is an important finding that the predictive biomarkers we identified for etanercept therapy differed from those for tocilizumab. These results further prove that individual patients tend to have a different response to each agent prescribed for the same disease condition and will discriminately react to some treatments more favorable than others.

In this analysis, we used DAS28-CRP to determine patients’ symptom levels. At the beginning of this study in 2008 the authors were not familiar with CDAI or SDAI assessments. Since DAS28-CRP appears to be well correlated with CDAI or SDAI we surmise that the prediction markers we detected may be applicable among all three assessments. Since IL-6 inhibitor blocks the production of CRP, it is considered ideal to avoid using DAS28-CRP for tocilizumab therapy. However, since our assessment involved no comparisons between tocilizumab and etanaercept, utilizing DAS28-CRP value did not significantly affect the predictive ability of the biomarkers identified. As previously mentioned we defined remission in this study as DAS28-CRP < 2.3 compared with the standard limit of 2.6. A preliminary comparison of DAS28-ESR and DAS28-CRP showed they produced relatively similar results (S1 Fig and S2 Table).

In this paper, we conducted a retrospective non-interventional cohort study in which we uncovered reliable biomarkers that can predict treatment response to tocilizumab and eternacept before patients undergo treatment for RA. These biomarkers may assist doctors to identify in advance patients who will not respond favorably to a treatment protocol thereby sparing patients from being treated with expensive and powerful agents that are not efficacious for them. It also allows individual RA patients to be matched with the anti-cytokine therapy that will be most effective for them or that will allow them to achieve their treatment target, indicating the personalized therapy in RA field.

This treatment strategy is line with the treat-to-target recommendation of EULAR and would facilitate personalized clinical medicine in the RA field. We believe our report is a critical first step and moving forward an interventional prospective study with a larger cohort should be utilized to confirm the predictive biomarkers that we have identified. In the meantime, examining these cytokine/chemokine and soluble receptor biomarkers before treating patients with biologic therapy is a pursuit that could be highly beneficial for RA patients and the doctors who treat them.

## Supporting Information

S1 Fig Comparison of DAS28-CRP and DAS28-ESR score. Plot of the baseline and week 16 DAS28-CRP and DAS28-ESR score in naïve and non-naïve tocilizumab treated patients.
(TIF)

S1 Table Multiple linear regression analysis of week 16 DAS28-CRP score for naïve patients in the tocilizumab with MTX treated group and the tocilizumamb only treated group using cytokine/chemokaine/soluble receptor levels (DOCX)

S2 Table Multiple linear regression analysis of week 16 DAS-ESR score using cytokine/chemokaine/soluble receptor levels (DOCX)
