# Supplementary material for: Pretreatment Prediction of Individual Rheumatoid Arthritis Patients’ Response to Anti-Cytokine Therapy Using Serum Cytokine/Chemokine/Soluble Receptor Biomarkers
Source: PLoS One. 2015 Jul 15;10(7):e0132055. doi: 10.1371/journal.pone.0132055 (PMC4503565; doi:10.1371/journal.pone.0132055)
Supplement: S1 Table — (DOCX) [file pone.0132055.s002.docx]

**S1 Table Multiple linear regression analysis of week 16 DAS28-CRP score for naïve patients in the tocilizumab with MTX treated group and the tocilizumamb only treated group using cytokine/chemokaine/soluble receptor levels**

|  | Biologic naïve patients treated with tocilizumab | | Biologic naïve patients treated with tocilizumab and MTX | |
| --- | --- | --- | --- | --- |
| Number of patients  (Female/Male) | n=29  (F/M:28/1) | | n=14  (F/M:14/0) | |
| R^2^ | 0.711 | | 0.891 | |
| p value | p<0.0001 | | P=0.015 | |
| Cytokine/Chemokine/soluble receptor | Estimate | p value | Estimate | p value |
| intercept | 9.91 | <.0001 | 3.19 | 0.250 |
| sgp130 | -3.63 | 0.049 | - | - |
| logIP-10 | -1.46 | 0.0030 | -1.60 | 0.073 |
| logIL-6 | ,.75 | 0.008 | 1.04 | 0.073 |
| logIL-8 | 2.41 | 0.004 | 7.03 | 0.011 |
| logEotaxin | -1.28 | 0.000 | -1.19 | 0.018 |
| logsTNFRI | -1.25 | 0.001 | -5.70 | 0.001 |
| logsTNFRII | - | - | 4.80 | 0.004 |
| logVEGF | - | - | -1.98 | 0.025 |
