# Supplementary material for: Pretreatment Prediction of Individual Rheumatoid Arthritis Patients’ Response to Anti-Cytokine Therapy Using Serum Cytokine/Chemokine/Soluble Receptor Biomarkers
Source: PLoS One. 2015 Jul 15;10(7):e0132055. doi: 10.1371/journal.pone.0132055 (PMC4503565; doi:10.1371/journal.pone.0132055)
Supplement: S2 Table — (DOCX) [file pone.0132055.s003.docx]

**S2 Table Multiple linear regression analysis of week 16 DAS-ESR score using cytokine/chemokaine/soluble receptor levels**

|  | Biologic naïve patients treated with tocilizumab | | Non-naïve patients treated with tocilizumab | |
| --- | --- | --- | --- | --- |
| Number of patients  (Female/Male) | n=45(F/M:42/3) | | n=37(F/M:31/6) | |
| R^2^ | 0.437 | | 0.486 | |
| p value | p=0.0003 | | p<0.0001 | |
| Cytokine/Chemokine/soluble receptor | Estimate | p value | Estimate | p value |
| intercept | 3.26 | <.0001 | 2.28 | 0.189 |
| sgp130 | -7.32 | 0.049 | -8.18 | 0.003 |
| logIP-10 | -0.89 | 0.003 | 0.97 | 0.083 |
| logIL-6 | 0.60 | 0.008 | - | - |
| logIL-8 | 3.17 | 0.004 | - | - |
| logEotaxin | -1.03 | 0.000 | - | - |
| logGM-CSF | - | - | -0.68 | 0.003 |
